# Enhancement of handwritten text recognition using AI-based hybrid approach

**DOI:** 10.1016/j.mex.2024.102654

**Published:** 2024-03-10

**Authors:** Supriya Mahadevkar, Shruti Patil, Ketan Kotecha

**Affiliations:** a PhD Research Scholar, Symbiosis Institute of Technology, Symbiosis International (Deemed University), Pune 412115, India; b Symbiosis Centre for Applied Artificial Intelligence (SCAAI), Symbiosis Institute of Technology, Symbiosis International (Deemed University), Pune 412115, India

**Keywords:** Handwritten text recognition, Long short-term memory (LSTM), Connectionist temporal classification (CTC), Machine learning, Deep learning, Etc, CNN +BiLSTM +CTC Hybrid approach using AI for Handwritten Text Recognition

## Abstract

Handwritten text recognition (HTR) within computer vision and image processing stands as a prominent and challenging research domain, holding significant implications for diverse applications. Among these, it finds usefulness in reading bank checks, prescriptions, and deciphering characters on various forms. Optical character recognition (OCR) technology, specifically tailored for handwritten documents, plays a pivotal role in translating characters from a range of file formats, encompassing both word and image documents. Challenges in HTR encompass intricate layout designs, varied handwriting styles, limited datasets, and less accuracy achieved.

Recent advancements in Deep Learning and Machine Learning algorithms, coupled with the vast repositories of unprocessed data, have propelled researchers to achieve remarkable progress in HTR. This paper aims to address the challenges in handwritten text recognition by proposing a hybrid approach. The primary objective is to enhance the accuracy of recognizing handwritten text from images. Through the integration of Convolutional Neural Networks (CNN) and Bidirectional Long Short-Term Memory (BiLSTM) with a Connectionist Temporal Classification (CTC) decoder, the results indicate substantial improvement. The proposed hybrid model achieved an impressive 98.50% and 98.80% accuracy on the IAM and RIMES datasets, respectively. This underscores the potential and efficacy of the consecutive use of these advanced neural network architectures in enhancing handwritten text recognition accuracy.

• The proposed method introduces a hybrid approach for handwritten text recognition, employing CNN and BiLSTM with CTC decoder.
• Results showcase a remarkable accuracy improvement of 98.50% and 98.80% on IAM and RIMES datasets, emphasizing the potential of this model for enhanced accuracy in recognizing handwritten text from images.

## Graphical abstract

**Figure:**
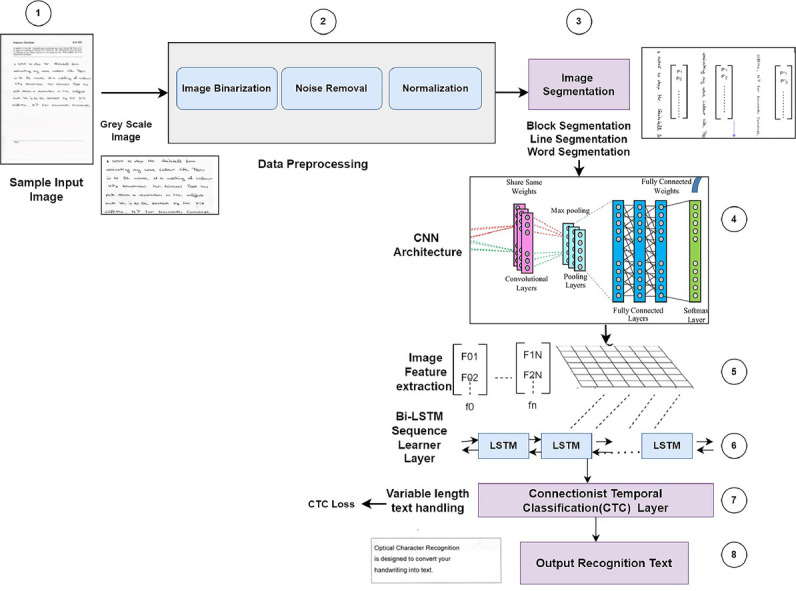

Specifications table

**Table:** 

Subject area:	Computer Science
More specific subject area:	*Computer Vision- Handwritten Text Recognition*
Name of your method:	*CNN +BiLSTM +CTC Hybrid approach using AI for Handwritten Text Recognition*
Name and reference of original method:	*NA*
Resource availability:	*Datasets link-*
	IAM-https://www.kaggle.com/datasets/naderabdalghani/iam-handwritten-forms-dataset
	RIMES- https://paperswithcode.com/dataset/rimes

## Introduction

Handwritten text recognition plays a vital role in numerous applications such as digitizing historical documents, transcribing handwritten notes, processing forms, and facilitating the efficient reading of handwritten materials in diverse domains. Enhancing the accuracy of handwritten text recognition makes vast amounts of handwritten data accessible and searchable, promoting efficient retrieval and analysis. This is especially relevant in research, education, and archival contexts.

HTR lies in its ability to bridge the gap between analog and digital worlds, making handwritten content more accessible, searchable, and usable across a wide range of applications and industries. So, there is a necessity and extensive opportunity for research and development in this domain to enhance the performance of handwritten text recognition.

This research manuscript presents details on the datasets utilized in the experimentation, the hybrid methodology proposed along with its workflow, the steps of the algorithm implementation, and the results, including a performance comparison with existing approaches.

The following are the major contributions and summary made by this study:

1. The authors proposed an efficient approach that unified the end-to-end trainable HTR system blended with a CTC decoder.
2. The proposed procedure adopts two state-of-the-art algorithms CNN and LSTM for training and the CTC decoder with WBS in the output recognition stage.
3. Authors have achieved state-of-the-art results by improving the Accuracy as 98.50% and Word Error Rate (WER) to 1.5%, 1.2%, respectively on the IAM dataset and RIMES dataset.
4. Detailed discussion of each step involved in a generic HTR process. It will be helpful for future readers to understand the whole process systematically.

## Method details

This section provides a detailed description of the proposed methodological process, datasets applied, and proposed algorithm steps leveraged for the handwritten text recognition experimentation.

### Datasets used

1. The IAM handwriting dataset includes the forms of handwritten text, which can be used for training and testing purposes of text recognition experiments. It corresponds to gray-scale images of English handwriting with a resolution of 300 dpi. We have used paragraph and line-level segmentation in this work with the train, test, and validation split. This dataset was organized to ensure a line recognition task independent of the writer, which means that each writer's handwriting is found only in a single subset. Then, image partitioning for the HTR system is 6161 for training, 900 for validation, and 1861 for testing [1]. Fig. 1 illustrates the randomly collected sample images from IAM and RIMES datasets, respectively.

**Fig. 1:**
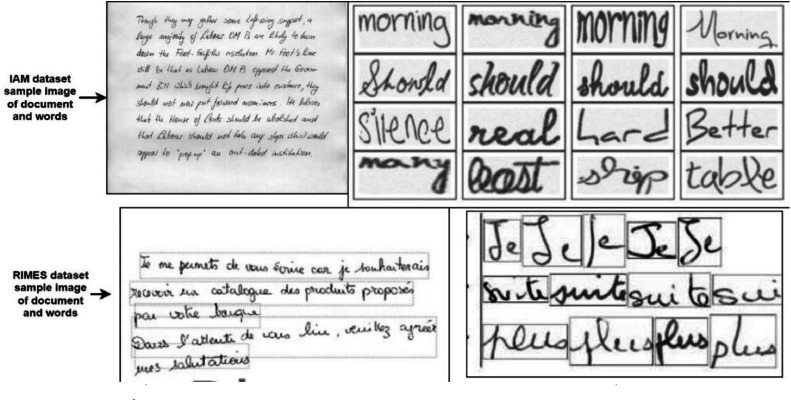
Sample input document and words from IAM and RIMES dataset.
2. The RIMES dataset is a collection of handwritten letters sent by individuals to company administrators. RIMES is a popular handwriting dataset composed of Gray-scale images of French handwritten text produced in the context of writing mail scenarios. The images have a resolution of 300 dpi. In the official split, there are 1500 pages for training and 100 pages for evaluation. The partitioning for the HTR system consists of 10,193 lines of text for training, 1133 for validation, and 778 for testing. As for the Spelling Corrector, 171,376 are for training and 19,041 for validation [2].

### Experimental setup and working flow-

A hybrid framework of CNN (Convolutional Neural Network), BiLSTM (Bidirectional Long Short-Term Memory), and CTC (Connectionist Temporal Classification) for handwritten text recognition involves combining these architectures to effectively recognize and transcribe text from images. Let's break down the working of this hybrid framework step by step:

#### 1. Convolutional neural network (CNN)

CNN is used for extracting hierarchical and spatial features from the input images.

Working Mechanism: The input to the CNN is a handwritten gray scale image from IAM and RIMES datasets.

• Convolutional Layers: The convolutional layers consist of filters that slide over the input image to capture local patterns and features. Each layer captures different levels of abstraction, allowing the network to learn low-level to high-level features.
• Pooling Layers: Pooling layers downsample the feature maps, reducing their spatial dimensions. This helps in retaining the most important features and reducing computational complexity.
• Flattening: The output feature maps are flattened to create a feature vector.

#### 2. Bidirectional long short-term memory (BiLSTM)

Sequential Context Modeling: BiLSTM is employed for modeling sequential dependencies in the feature sequences obtained from the CNN.

The feature vector obtained from the CNN serves as the input sequence for the BiLSTM.

• Bidirectional LSTM: Bidirectional LSTMs process the input sequence in both forward and backward directions. This allows the model to capture dependencies in both the past and future contexts.
• Hidden States: The hidden states of the BiLSTM encode contextual information at each time step, incorporating information from both directions.
• Output Sequence: The output sequence from the BiLSTM represents a contextualized and enriched version of the input feature sequence.

#### 3. Connectionist temporal classification (CTC)

Alignment and Transcription: CTC is applied for handling the alignment between the sequential output of the BiLSTM and the ground truth transcriptions.

Working Mechanism:

• Prediction Layer: The output sequence from the BiLSTM is passed through a prediction layer with SoftMax activation. The SoftMax output represents the probabilities of different characters at each time step.
• CTC Loss Calculation: The CTC loss is calculated by comparing the predicted sequence with the ground truth transcription. CTC loss accounts for variable-length alignments and allows the model to learn to align the predictions with the ground truth.
• Objective of Training process: During training, the model aims to minimize the CTC loss, adjusting the parameters of both the CNN and BiLSTM.
• Integration of CNN, BiLSTM, and CTC: The CNN, BiLSTM, and CTC components are combined into a unified architecture. The output of the CNN serves as the input to the BiLSTM, and the sequential output of the BiLSTM is used for transcription through the CTC mechanism.
• Training Process: The entire hybrid model is trained end-to-end. The loss is backpropagated through both the CNN and BiLSTM components. The model learns to extract spatial features with CNN, model sequential dependencies with BiLSTM, and align predictions with CTC.
• Inference: During inference, a trained model is used to transcribe handwritten text images. The model processes an image through the CNN-BiLSTM pipeline, and the CTC decoding is applied to obtain the final transcriptions.
• Advantages: Spatial and Sequential Information: CNN captures spatial features, useful for recognizing characters and patterns. BiLSTM models sequential dependencies, essential for understanding the context of characters in a sequence.
• End-to-End Training: The entire framework is trained in an end-to-end manner, allowing the model to learn effective representations at both the spatial and sequential levels.
• Variable-Length Handling: CTC facilitates the handling of variable-length sequences during training and decoding.
• Effective Text Recognition: The combination of CNN, BiLSTM, and CTC has proven effective for handwritten text recognition tasks, achieving state-of-the-art performance on datasets like IAM and RIMES.

In summary, the hybrid framework integrates spatial and sequential information, leveraging the strengths of CNN, BiLSTM, and CTC to accurately recognize and transcribe handwritten text from images. This approach has demonstrated success in handling the challenges posed by variable-length sequences and diverse handwriting styles in datasets like IAM and RIMES.

## Results and discussion

### Step 1: preprocessing and segmentation

Before the necessary recognition algorithms are applied, a variety of operations must be carried out on the handwritten text data as part of the pre-processing of handwriting. This phase tackles the issues of data normalization, inconsistency elimination, and dimensionality reduction. It generates a data set more suited for the data segmentation found in the image format [3]. Two main phases are involved in implementing pre-processing: line segmentation and word segmentation utilizing the OpenCV library. Input images from IAM and RIMES datasets are in grayscale format. During the pre-processing phase, the paragraphs from IAM dataset are primarily transformed into lines and then into words. Words are retrieved and the images used are grayscale throughout this process. It is accomplished efficiently with OpenCV. The pre-processing phase facilitates faster text recognition. Further gray scale images transformed into an inverse binary image and dilated. As transformation of a grayscale image into an inverse binary image followed by dilation is a common preprocessing step in image processing, particularly in tasks related to document analysis and handwritten text recognition.

#### 1. Image normalization

Image normalization is the process of transforming the grayscale values of an image to enhance its contrast, reduce variations in lighting, and improve its overall quality.

Importance: Normalization standardizes the appearance of images, making them more suitable for processing algorithms and improving the robustness of the recognition system to variations in input.

Techniques:

• Brightness Correction: Adjust the brightness of the image to ensure consistent illumination across different samples.
• Scaling and Resizing: Resize the images to a standard size to ensure consistency and facilitate processing.

Following are the working steps:

• The grayscale image from IAM and RIMES obtained from the binarization step.
• Then contrast enhancement techniques applied to improve the visibility of text regions and reduce variations in lighting.
• Next step is to adjust the brightness of the image if necessary to ensure consistent illumination. Resize the images to a standard size suitable for processing, typically scaling them to a specific resolution. In IAM and RIMES datasets, image normalization enhances the quality of handwritten text images, making them more legible and improving the performance of segmentation and recognition algorithms.

#### 2. Image binarization

It is the process of converting a grayscale image into a binary image, where each pixel is either classified as foreground text or background.

Importance: Binarization simplifies the subsequent processing steps by reducing the complexity of the image and isolating the text from the background.

Techniques:

• Global Thresholding: A single threshold value is applied to the entire image to classify pixels as foreground or background. Common methods include Otsu's method and simple thresholding based on intensity levels.
• Adaptive Thresholding: Different threshold values are applied to different regions of the image based on local characteristics, which is beneficial for images with non-uniform lighting conditions or varying backgrounds.

Implementation Steps:

• Load the grayscale image from the input dataset.
• Choose an appropriate binarization technique based on the characteristics of the dataset and the images.
• Apply the chosen binarization technique to obtain a binary image representation.
• Fine-tune the thresholding parameters if necessary to achieve optimal results.

In IAM and RIMES datasets, adaptive thresholding applied which is helpful to separate handwritten text from the background, facilitating subsequent segmentation and recognition tasks.

#### 3. Dilation operation

Dilation is a morphological operation that enhances or expands regions in an image. It involves sliding a structuring element (a small matrix or kernel) over the image and setting the value of each pixel in the result to the maximum value of the pixels in the neighborhood defined by the structuring element. b. Purpose of Dilation in Handwritten Text Recognition:

Dilation helps connect broken or faint parts of the text, making the text regions more robust and prominent for subsequent analysis. It is particularly useful when the handwritten text might have gaps or variations in stroke thickness.

#### 4. Segmentation process

For line and word segmentation OpenCV computer vision library with segmentation techniques are applied.

##### Steps followed for line segmentation

• Load the input grayscale image using imread function.
• Apply thresholding to create binary image.
• Perform morphological operations to enhance the text regions.
• Find contours to identify individual text lines and extract individual lines by cropping bounding rectangles around contours.

##### Steps followed for word segmentation

• Apply thresholding and morphological operations. Then identify the contours to detect individual text words.
• Extract individual words by cropping bounding rectangles around contours.

In summary, Once the binary image's contours are located, they are applied using bounding boxes and stored separately as line images. The text lines that are produced as the result of line segmentation are divided into distinct words during the word segmentation process. Additionally, advanced techniques based on deep learning or text detection algorithms can also be explored in future for better accuracy in segmentation process.

### Step 2: training, validation, and testing

An 80:20 split of the datasets is made for testing and training. Eighty percent of the IAM word image dataset and the RIMES dataset—which is further divided into two for training and validation—are used to train the model. Two distinct training-validation ratios are used to train the model, and the remaining 20% of the dataset is used for further testing. The handwritten custom images and paragraph images from the IAM dataset are randomly selected and preprocessed.

### Step 3: text recognition

The input image's text is then recognized using the trained neural network model. The CNN layers are initially applied to the input pictures. The CNN layers receive the input image from IAM and RIMES dataset. These layers are trained to identify important features in the image. Every layer is made up of three processes. Initially, the non-linear RELU function is used after the convolution process. Ultimately, a pooling layer condenses image areas and generates a reduced input size. Every time-step in the feature sequence has 256 features. Pooling layers are a crucial component in Convolutional Neural Networks (CNNs), contributing to the reduction of spatial dimensions and the extraction of hierarchical features. In the context of handwritten text recognition using a hybrid approach of CNN-BiLSTM and Connectionist Temporal Classification (CTC) on datasets like RIMES and IAM, pooling layers play a role in feature extraction and computational efficiency. The softmax layer in a neural network, particularly in the context of handwritten text recognition, is used to convert the network's raw output into a probability distribution over a predefined set of classes or characters. In the case of text recognition, each class typically represents a possible character (e.g., letters, digits, special symbols). The softmax function normalizes the raw output scores (logits) into probabilities, and each output neuron's activation represents the probability of the corresponding class. This is done independently for each position in the output sequence (each character in the recognized text).

**Fig. 2:**
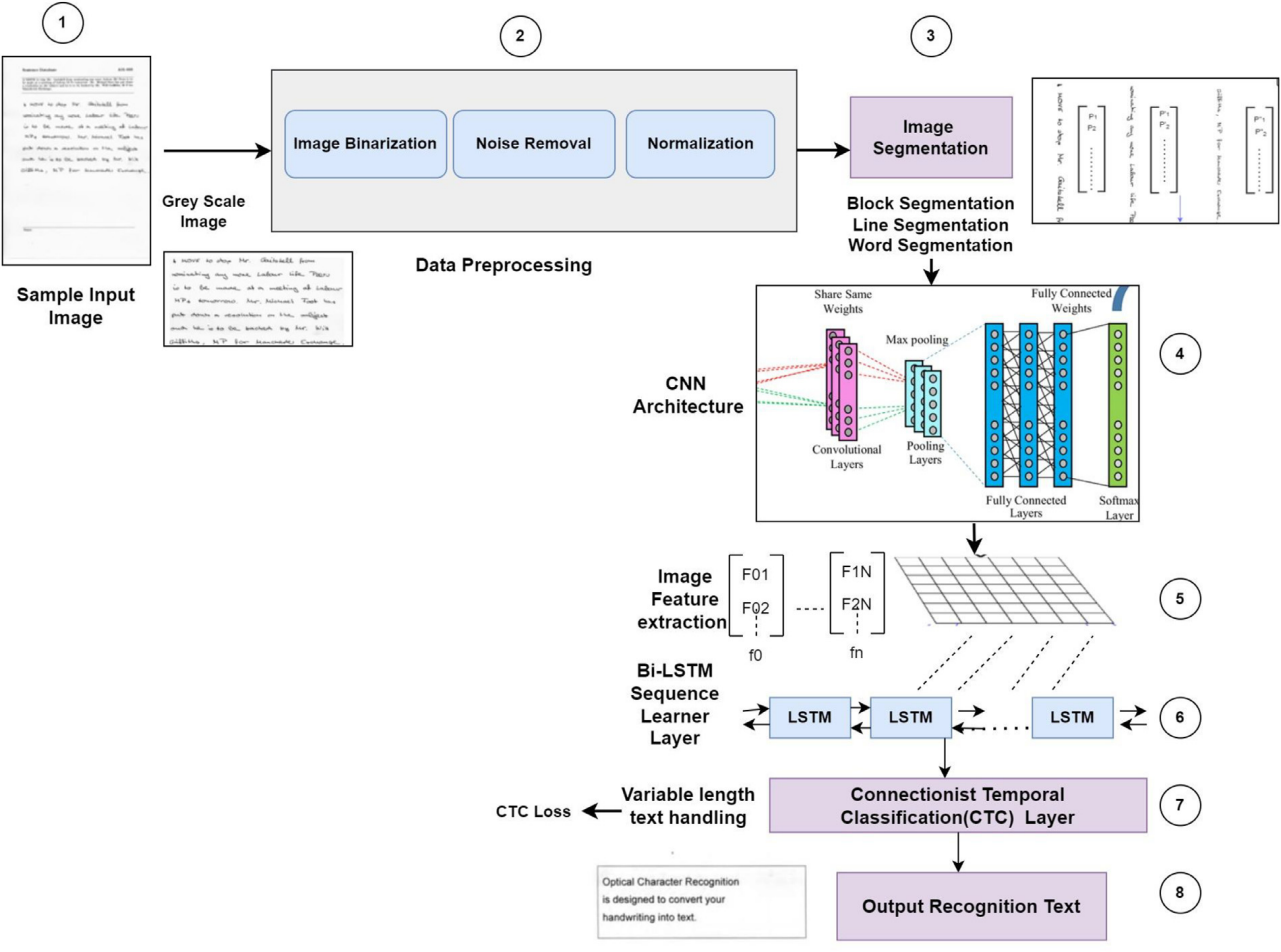
Workflow and performance evaluation of Handwritten text recognition.

For this experimentation authors have used a part learnt from the IAM and RIMES dataset respectively. A 32-character sequence is produced by each CNN layer. Each entry that is subsequently processed by the LSTM layers has 256 features. The CTC receives the RNN layers' output to decode the output text. Fig. 2 shows the stepwise proposed workflow of how handwritten text recognition is performed on publicly available datasets.

### Step 4: results and evaluation-

The current study is assessed using cutting-edge evaluation criteria including WER, CER, and accuracy. Fig. 4 displays the performance evaluation of proposed technique on IAM and RIMES datasets in terms of accuracy. Whereas Fig. 5 shows the word error rate occurred in proposed framework in case of IAM and RIMES datasets. Based on the words that were properly identified over the whole data corpus, the accuracy is calculated. Based on the Levenshtein Distance (Ld) between the projected text (p) and ground truth (g), the CER is calculated. The WER and CER are identical; however, as stated in Eq. (1), the evaluation of the WER takes place at the word level as opposed to the character level.

(1) Accuracy=Correctlyrecognizedsetofwords/(TotalSetofwordsfromcorpus)*100

(2) CER=(sub+ins+delt)/n_total

Where,

The number of character substitutions is sub.

The number of character insertions is ins.

The number of deletions concerning the anticipated text is delt.

The entire number of characters in the real string is n_total.

Accuracy, WER and CER are the major performance measure in the case of handwritten text recognition from images. Using proposed hybrid approach Accuracy and WER evaluated on IAM and RIMES datasets. Following are the steps for handwritten training algorithm implemented.

### Proposed HTR model training algorithm

Input: Paragraph batch images PI, and ground Truth Gl with lines Gl1,

Gl2…Gl1 and data corpus (IAM, RIMES)

Result: Training using NN backpropagation and Evaluation Metrics on given

Dataset

1. Initialize main ();
2. Params=init_Params();
3. setDevice(Params);
4. Dataset=load_Dataset(Params);
5. *I*=preprocess Dataset (input dataset);

Data normalization, Binarization, Dilation, Noise removal
Segmentation: line, word, character

6. Training the model on data samples using hybrid architecture

CNN and BiLSTM;

7. predict=Testing ();
8. predict=SoftMax(predict);
9. Transcript=Concat (Transcript, WBS(predt, Corptext)); //CTC Decoder
10. CER, WER= Accuracy (Transcript, ground truth)

Performance evaluation done on transcription and ground truth results.

## Method validation

**Fig. 3:**
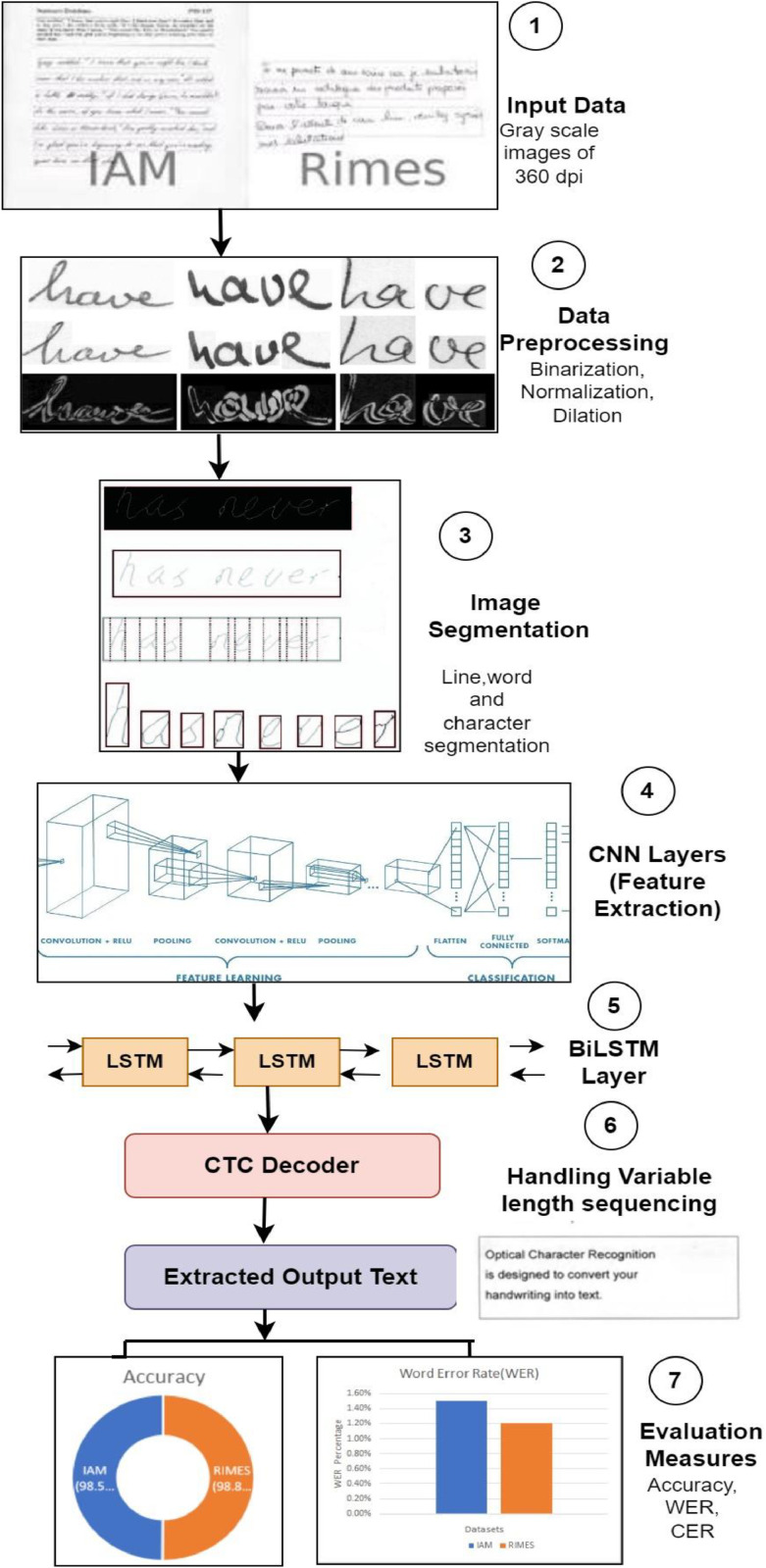
Performance Evaluation of Handwritten Text Recognition process.

**Fig. 4:**
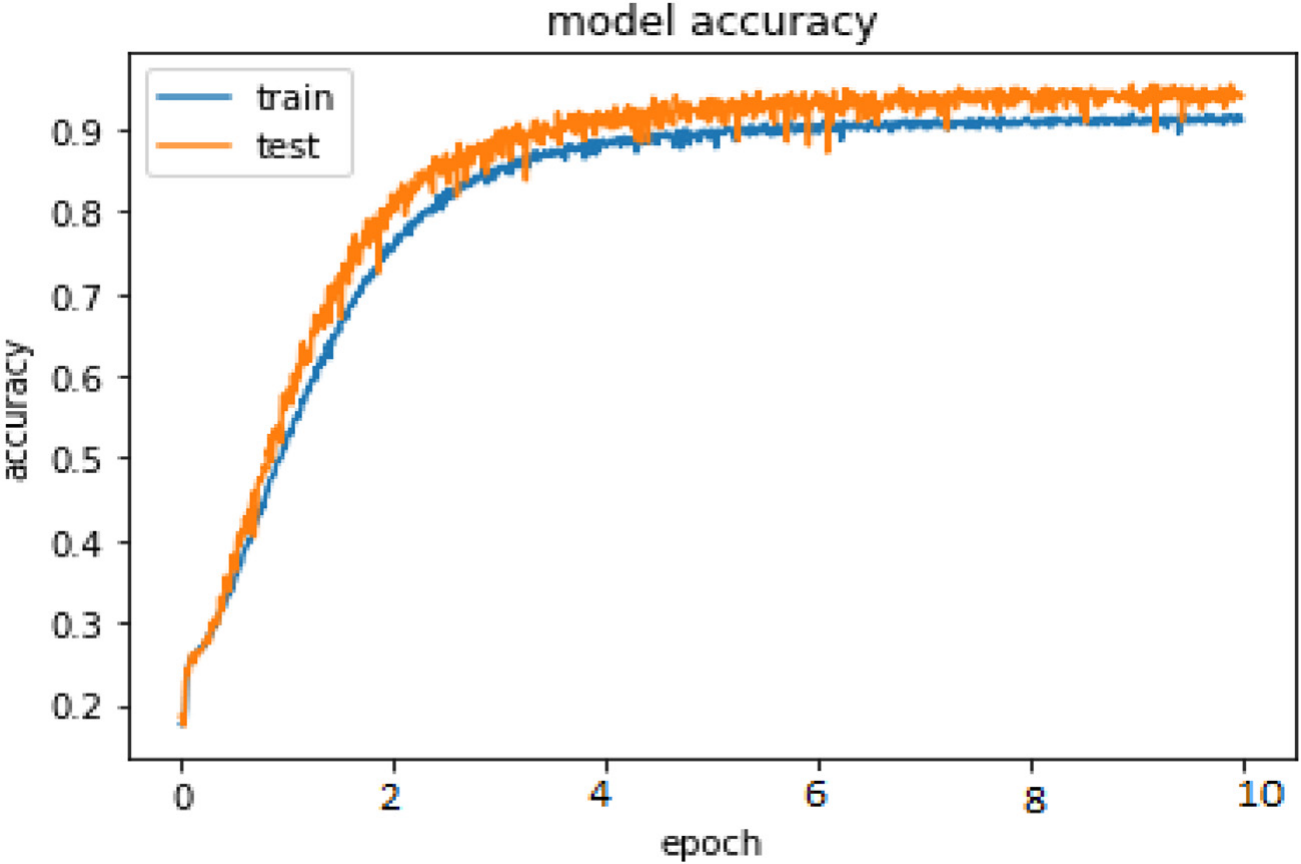
Experimental results of training and validation accuracy on IAM dataset.

**Fig. 5:**
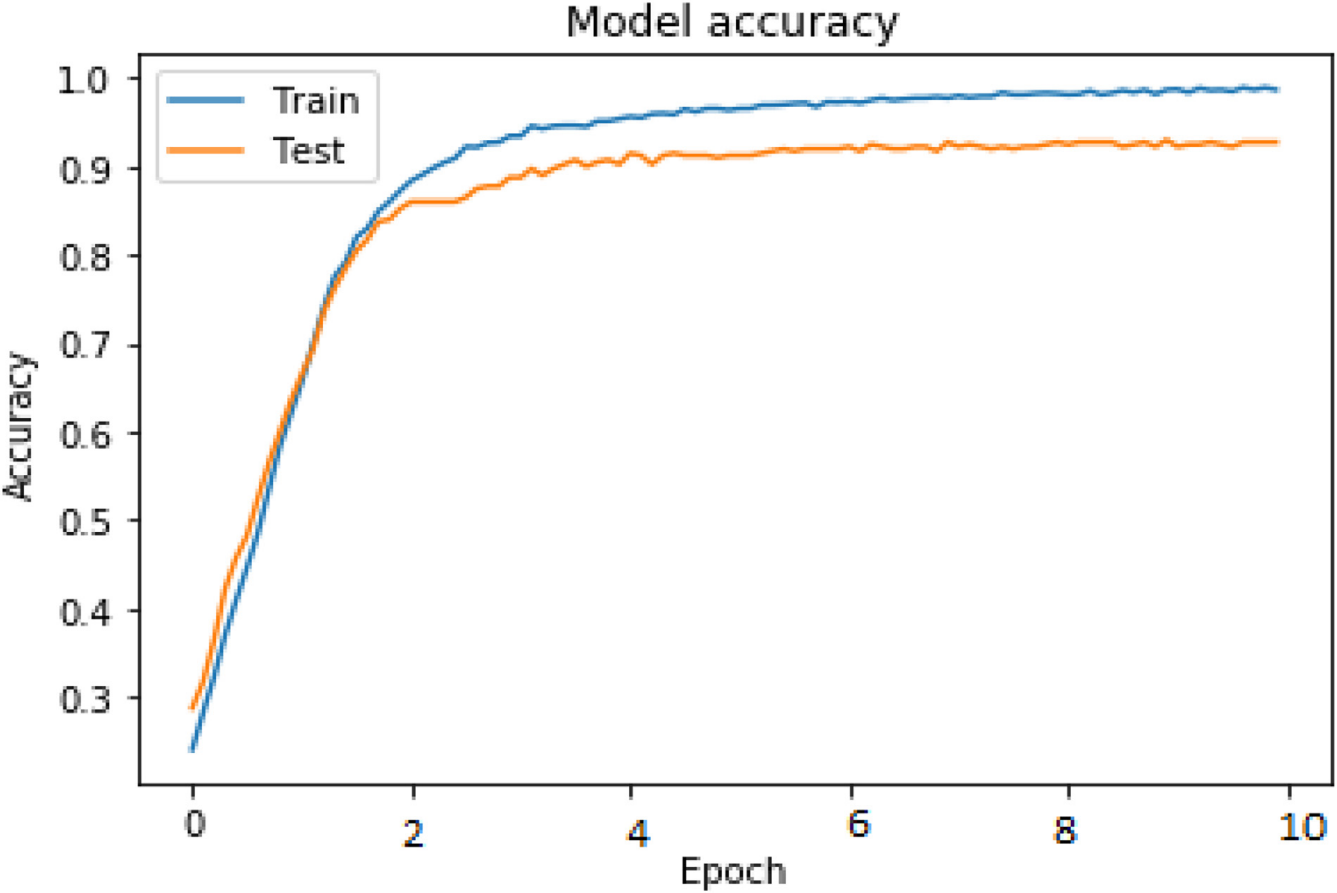
Experimental results of training and validation accuracy on RIMES dataset.

**Fig. 6:**
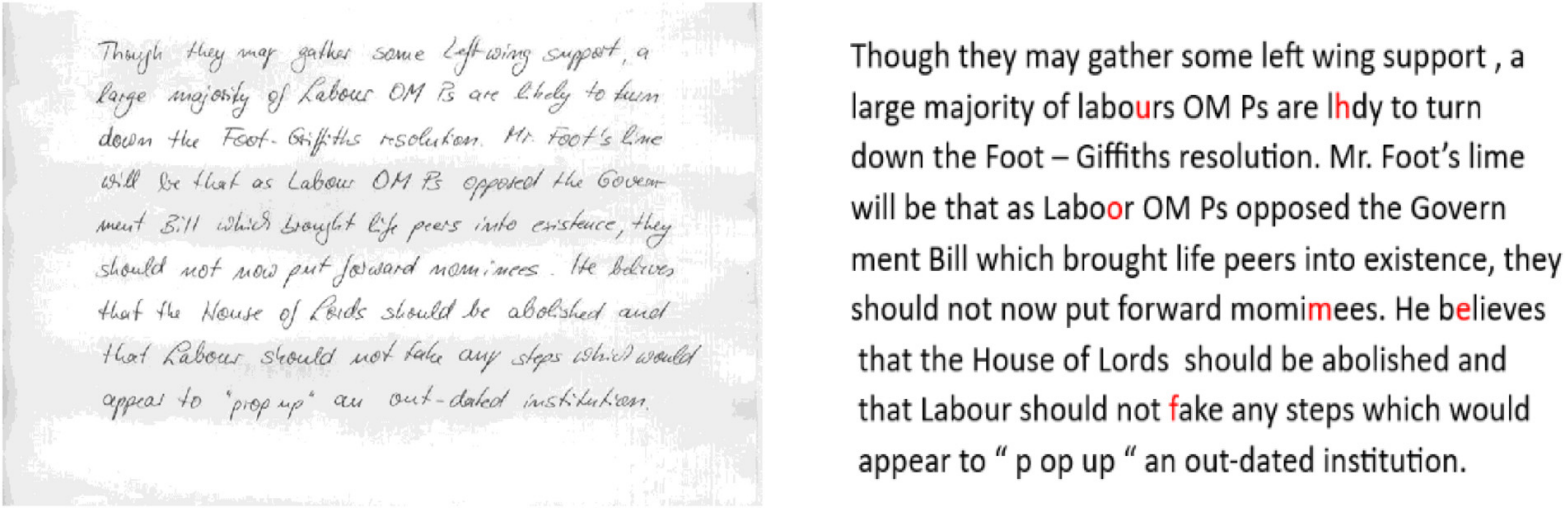
Sample input data image and prediction result of IAM dataset.

**Fig. 7:**
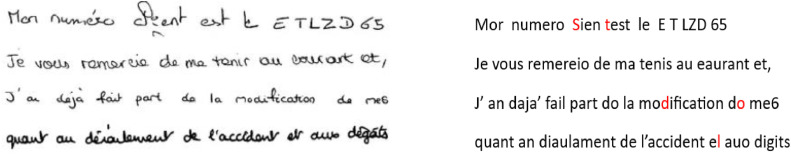
Sample input data image and prediction result of RIMES dataset.

**Fig. 8:**
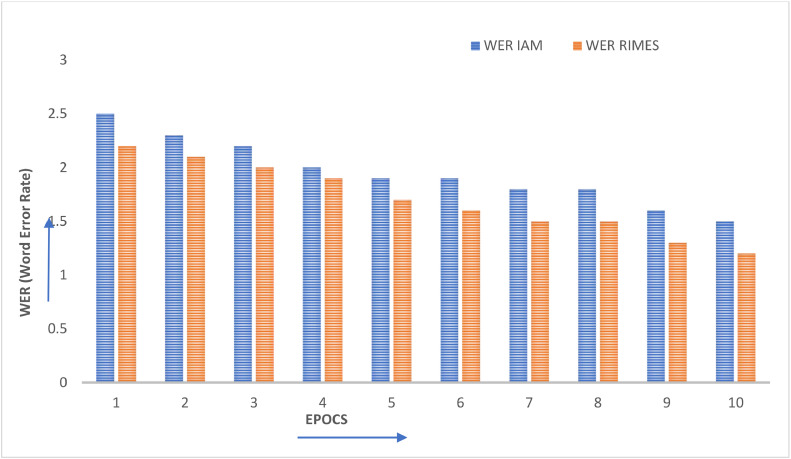
Word Error Rate (WER) of IAM and RIMES datasets on proposed technique.

First, as seen in Fig. 2, we systematically designed a hybrid technique for handwriting test recognition using CNN & BiLSTM with a CTC decoder. The handwritten text recognition results from the IAM and RIMES datasets, respectively, are trained and tested using an AI-based hybrid model that has undergone image binarization and normalization techniques of preprocessing. There are several ways to recognize handwritten text, however each approach has drawbacks since handwritten text is often non-trivial. For this reason, the most efficient approach with a suitable technique is applied. Table 1 shows the experimental results of the proposed hybrid approach on IAM and RIMES datasets in terms of Accuracy and WER. It displays the overall performance improvement in terms of Accuracy and WER on IAM and RIMES datasets compared with the existing literature. In the case of proposed hybrid approach compared with existing approaches overall accuracy achieved on IAM and RIMES datasets is improved, and word error rate decreased. There is potential improvement in accuracy achieved by proposed hybrid methodology on IAM and RIMES datasets respectively. Fig. 3 describes step by step process from the initial step till the final performance evaluation. Where the initial phase describes the input dataset loading, then the preprocessing is performed on data. On preprocessed data the model gets train using proposed hybrid model. At the end CTC decoder extracts the output text. Fig. 4, Fig. 5 show the training and validation accuracy of IAM and RIMES datasets respectively. Further Fig. 6, Fig. 7 illustrate the sample image and recognition output from IAM and RIMES datasets using proposed approach. Fig. 8 displays the word error rate occured in this experimentation using proposed hybrid approach on IAM and RIMES datasets.

**Table 1:** Experimental results on IAM and RIMES dataset [2], [3], [4], [5], [6], [7], [8], [9], [10].

Ref	Dataset Used	Methodology Used	Experimental Results (Accuracy)	WER	CER
[2]	MIMO and UCSD	CNN, RNN	86.37–90.5%	–	–
[4]	IAM, RIMES	CNN, LSTM	95%	–	–
[5]	Chars74k	FLM	92–95%	–	–
[6]	Self-built Shui character	CNN	93.3%	–	–
[7]	IAM and Customized Handwritten	CNN, RNN, CTC	98%	–	–
[8]	IAM, RIMES	RNN	–	18.0%	4.7%
				13.1%	3.3%
[9]	IAM, RIMES	CNN+RNN	–	12.61%	4.88%
				7.04%	2.32%
[10]	IAM Dataset	Vertical Attention Network	–	12.7%	6.2%
Proposed Approach	IAM and RIMES	CNN, BiLSTM, CTC	98.55%,98.80%	1.5%,1.2%	–

## Conclusion

By using CNN and BiLSTM to train the dataset sequentially, an adaptive approach for offline sentence recognition has been proposed in this system. Instead of being fed into the NN model layers for recognition, the input paragraph images are first pre-processed using OpenCV contour algorithms and divided into line images. From there, the line images are processed into word images. The BiLSTM layers carry out further processing on the CNN layers' output. The CTC receives the BiLSTM layers' output to decode the output text. The outcomes demonstrate the potential for using CNN and BiLSTM with a CTC decoder in order, which improved the accuracy of handwritten text recognition to 98.55% and 98.80%, respectively. By utilizing hybrid datasets, experimenting with various activation functions, and adding more neural network layers to the work, we hope to improve it in the future. Our next goal is to improve the work even further by incorporating online recognition and translating it into other languages. We can also encourage the system to identify characters that are broken or have low quality.

## Ethics statements

Our work is not related to social media data, animal subject or human subject related. Data which we have used for experimentation is publicly available.

## CRediT authorship contribution statement

**Supriya Mahadevkar:** Conceptualization, Visualization, Software. **Shruti Patil:** Validation, Data curation, Writing – original draft, Methodology, Software, Validation. **Ketan Kotecha:** Supervision, Investigation.

## Declaration of competing interest

The authors declare that they have no known competing financial interests or personal relationships that could have appeared to influence the work reported in this paper.

## Data Availability

No data was used for the research described in the article.
